# The combined effect of mammographic texture and density on breast cancer risk: a cohort study

**DOI:** 10.1186/s13058-018-0961-7

**Published:** 2018-05-02

**Authors:** Johanna O. P. Wanders, Carla H. van Gils, Nico Karssemeijer, Katharina Holland, Michiel Kallenberg, Petra H. M. Peeters, Mads Nielsen, Martin Lillholm

**Affiliations:** 1 Julius Center for Health Sciences and Primary Care, University Medical Center Utrecht, P.O. Box 85500, 3508 GA Utrecht, The Netherlands; 2 Department of Radiology and Nuclear Medicine, Radboud University Medical Center, Geert Grooteplein 10, 6525 GA Nijmegen, The Netherlands; 3 Department of Computer Science, University of Copenhagen, Universitetsparken 5, DK-2100 Copenhagen, Denmark; 4 Biomediq A/S, Fruebjergvej 3, 2100 Copenhagen, Denmark; 5 MRC-PHE Centre for Environment and Health, Department of Epidemiology and Biostatistics, School of Public Health, Imperial College London, St. Mary’s Campus, Norfolk Place W2 1PG, London, UK

**Keywords:** Volumetric mammographic breast density, Texture pattern scores, Breast cancer risk

## Abstract

**Background:**

Texture patterns have been shown to improve breast cancer risk segregation in addition to area-based mammographic density. The additional value of texture pattern scores on top of volumetric mammographic density measures in a large screening cohort has never been studied.

**Methods:**

Volumetric mammographic density and texture pattern scores were assessed automatically for the first available digital mammography (DM) screening examination of 51,400 women (50–75 years of age) participating in the Dutch biennial breast cancer screening program between 2003 and 2011. The texture assessment method was developed in a previous study and validated in the current study. Breast cancer information was obtained from the screening registration system and through linkage with the Netherlands Cancer Registry. All screen-detected breast cancers diagnosed at the first available digital screening examination were excluded. During a median follow-up period of 4.2 (interquartile range (IQR) 2.0–6.2) years, 301 women were diagnosed with breast cancer. The associations between texture pattern scores, volumetric breast density measures and breast cancer risk were determined using Cox proportional hazard analyses. Discriminatory performance was assessed using c-indices.

**Results:**

The median age of the women at the time of the first available digital mammography examination was 56 years (IQR 51–63). Texture pattern scores were positively associated with breast cancer risk (hazard ratio (HR) 3.16 (95% CI 2.16–4.62) (*p* value for trend <0.001), for quartile (Q) 4 compared to Q1). The c-index of texture was 0.61 (95% CI 0.57–0.64). Dense volume and percentage dense volume showed positive associations with breast cancer risk (HR 1.85 (95% CI 1.32–2.59) (*p* value for trend <0.001) and HR 2.17 (95% CI 1.51–3.12) (*p* value for trend <0.001), respectively, for Q4 compared to Q1). When adding texture measures to models with dense volume or percentage dense volume, c-indices increased from 0.56 (95% CI 0.53–0.59) to 0.62 (95% CI 0.58–0.65) (*p* < 0.001) and from 0.58 (95% CI 0.54–0.61) to 0.60 (95% CI 0.57–0.63) (*p* = 0.054), respectively.

**Conclusions:**

Deep-learning-based texture pattern scores, measured automatically on digital mammograms, are associated with breast cancer risk, independently of volumetric mammographic density, and augment the capacity to discriminate between future breast cancer and non-breast cancer cases.

**Electronic supplementary material:**

The online version of this article (10.1186/s13058-018-0961-7) contains supplementary material, which is available to authorized users.

## Background

Many countries have a breast cancer screening program [1]. The intention of these programs is to find breast cancers at an early stage, to increase the chance of successful treatment and to prevent premature mortality [2, 3]. Although most screening programs have been shown to decrease breast cancer mortality [4], the programs do not work equally well for all women. It is well-known that women with more fibroglandular breast tissue (dense tissue) have a lower probability that a cancer, if present, is detected through mammographic screening: the screening sensitivity is lower in women with dense breasts [5–13]. High mammographic density does not only lower mammographic screening sensitivity, it is also a well-known breast cancer risk factor [14, 15]. Therefore, the possibility of developing more personalized screening, taking mammographic density and breast cancer risk into account, is being discussed widely [16, 17]. In the USA, legislation enforces physicians to inform a woman of her mammographic density after mammographic screening. Breast density legislation is now in place in 36 states. Depending on her mammographic density, a woman can choose to be screened with another imaging modality, like ultrasound (US) or magnetic resonance imaging (MRI), in addition to mammography [16].

Besides mammographic density, mammographic texture patterns have also been shown to be associated with breast cancer risk, and also to improve breast cancer risk segregation in addition to area-based mammographic density [18–21]. These texture patterns characterize the spatial distribution of parenchymal tissue in the breast. Examples of radiographic features of texture patterns are, for example, co-occurrence features, which take into account the pixel intensities or gray-levels of neighboring pixels in different directions; run-length features, which characterize the coarseness of the texture patterns by determining the length of consecutive pixels with the same pixel intensity in linear directions; structural features, which characterize the tissue complexity and variations in gray level between a specific pixel and its neighboring pixels; and multi-resolution or spectral features, which use frequency transforms, like Fourier or wavelet, to capture texture structures that are repeatedly found in a mammogram [19].

All these texture features are manually designed and selected and will only capture mammographic, risk-prone patterns to the extent the feature designs were relevant. This problem is normally overcome by initially using large banks of potential features but only maintaining the informative ones in the final classification system [22, 23]. However, more modern texture quantification methods based on deep learning address this challenge in a more principled, domain, and task-specific way [24]. Here, features are not designed but are learned from the domain data as part of training of the overall classification system. In its simplest form, as those features that best describe the domain (e.g., mammographic images) and in a more complex form as the features that best describe the domain and simultaneously contribute optimally to the task at hand (e.g., cancer risk). It has been suggested that the methods developed with deep learning have a better ability to quantify breast cancer risk compared to methods based on manually designed and selected texture features [24].

Therefore, the aim of this study was to determine the association between a previously developed deep-learning-based texture score [24] alone and in combination with automatically measured volumetric mammographic density and breast cancer risk, and their ability to segregate future breast cancer cases from non-breast cancer cases in a “new” dataset. This dataset consists of a consecutive series of unprocessed digital mammograms of a breast cancer screening population in whom mammograms are prospectively collected.

## Methods

### Study population

In the Netherlands, women aged 50–75 years have been invited for mammographic breast cancer screening every other year from 1989 and onwards. Approximately 80% of the women attend the screening program [25]. Since 2003 the transition from analog to digital mammography gradually took place, starting at one screening unit (Preventicon screening unit, Utrecht, The Netherlands) and in 2010 the transition was complete. For this study, all women were included who had one or more digital mammographic screening examinations at the Preventicon screening unit between 2003 and 2011. There are five screening regions in the Netherlands that follow the exact same procedures. The Preventicon screening unit is part of the Foundation of Population Screening Mid-West region. Women consent to their data being used for evaluation and improvement of the screening, by participating in the Dutch breast cancer screening program, unless they have stated otherwise.

The research ethics committee of the Radboud University Nijmegen Medical Centre declared that this study does not fall within the remit of the Medical Research Involving Human Subjects Act. Therefore, this study could be carried out (in The Netherlands) without approval by an accredited research ethics committee.

### Data collection

We selected each woman’s first unprocessed (raw) digital mammography examination. All mammograms were taken using Lorad Selenia DM systems (Hologic, Danbury, CT, USA). During the first examination in the screening program, both craniocaudal (CC) and mediolateral oblique (MLO) views are always acquired. In subsequent rounds the MLO is the standard view and an additional CC view is taken only when indicated (e.g., visible abnormality, high mammographic density). Information during follow up was obtained through the screening registration system and through linkage with the Netherlands Cancer Registry to obtain complete information on both screen-detected and interval breast cancers. Screen-detected breast cancers were defined as breast cancers diagnosed on the basis of diagnostic work-up of an “abnormal” screening examination. Interval breast cancers were defined as breast cancers diagnosed within 24 months after a screening examination that did not lead to recall (negative mammogram), and before the next scheduled screening examination. The median time between the first available digital screening mammogram and breast cancer diagnosis was 3.7 years (IQR 2.0–4.3, minimum 0.1 years, maximum 7.9 years) for screen-detected breast cancers and 2.2 years (IQR 1.1–3.9, minimum 0.1 years, maximum 9.6 years) for interval cancers. Both invasive and ductal carcinoma in situ breast cancers were used for analyses.

We excluded all screen-detected breast cancer cases that were diagnosed based on the first digital screening examination, to minimize the number of breast cancer cases in the study that were diagnosed based on the same mammogram as was used for breast density and texture score assessment.

The texture measure used in this study was previously developed using a selection of women with and without breast cancer who had one or more digital mammographic screening examinations at the Preventicon screening unit between 2003 and 2011 [24]. Therefore, we also excluded all women whose mammograms were used to train the texture measure used in this study, to ensure an independent validation.

The data were obtained through the registry of a breast cancer screening program in which mammograms are routinely collected. Therefore, besides age, no additional information was available about the women.

### Volumetric mammographic density assessment

Absolute dense volume (DV) and percentage dense volume (PDV) were automatically assessed from unprocessed mammograms of the left and right breasts, using Volpara Density (version 1.5.0, Volpara Health Technologies, Wellington, New Zealand) [26]. We used the mean of the left and right MLO views, since this is the routinely acquired view and CC views were not available for all women. In this way, we ensured that mammographic density was assessed in the exact same way in all participants.

### Mammographic texture assessment

The deep-learning-based mammographic texture-based risk assessment was calculated from unprocessed mammograms using prototype software by Biomediq A/S as described by Kallenberg et al. [24]. The deep-learning framework was a 5-layer convolutional neural network that maps mammographic patches to a cancer risk score when trained as described below. The first four layers were three convolutional and one pooling layer. These layers learned mammographic features (mammographic structure/texture) of decreasing size and increasing level of abstraction. The initial three layers were trained in an unsupervised fashion: they learn features that describe mammographic structure independent of cancer risk. The final two layers (the last convolution layer and the final 5th Softmax classification layer) were trained in a supervised fashion using the features encoded in the previous layers as the starting point. The weights of these final layers were optimized to distinguish between patches from breasts without cancer diagnosis (at both baseline and follow up) and patches from breasts that were without diagnosis at baseline but were diagnosed with breast cancer at follow up. The implication of this is that the network was trained to score cancer risk realized as the probability that a patch originates from a breast with cancer-prone mammographic texture/structure. Further technical/mathematical details of texture methodology can be found in the article of Kallenberg et al. [24].The training dataset described subsequently corresponds to the dataset named “Dutch Breast Cancer Screening Dataset” in that same article [24]. For the purposes of this study, the deep-learning framework was trained on a subset of the Preventicon data consisting of 394 cancer cases and 1182 healthy controls - 3 controls per case, matched on age and acquisition date. The cancer cases included 285 screen-detected cancers and 109 interval cancers. For screen-detected cancers, the cases were represented by the contralateral view at the time of diagnosis. For interval cancers, the cases were represented by the contralateral view from the screening visit immediately prior to diagnosis. The laterality distribution of the controls was sampled to match that of the cases.

The left and right MLO views in the remaining independent validation subset of the Preventicon cohort were scored for texture-based risk using the framework above. The texture score for a single screening visit was obtained as the average of the left and right MLO texture risk scores. This scoring was performed such that both software and operator were fully blinded to cancer outcome during scoring. For each MLO view, the software extracted 500 randomly sampled patches within the fully compressed part of the breast tissue. To identify the fully compressed part of the breast, the geometry of the uncompressed breast is modelled as a semi-sphere, as has been proposed in the works of Highnam and Brady [27]. According to this model, the boundary between the fully compressed and the uncompressed part of the breast is found at those locations within the breast where the distance to the skin edge equals half the height of the breast. Each patch was scored for cancer risk using the trained deep-learning framework described above and the resulting texture score for a single view was obtained as the average of the 500 patch-based risk scores.

**Fig. 1:**
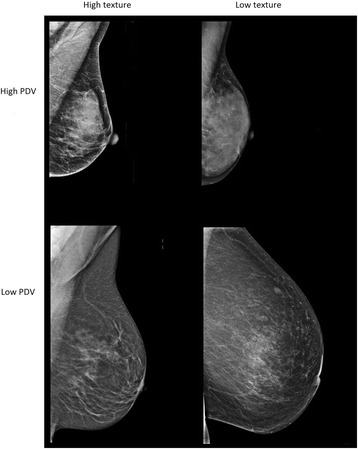
High and low texture and high and low density mammograms. Mammogram combinations of low and high texture patterns scores and percentage dense volume (PDV): high PDV (14%) and high texture (0.58) and high PDV (19%) and low texture (0.47) scores (top); low PDV (5%) and high texture (0.51) and low PDV (4%) and low texture scores (0.44) (bottom)

An example of mammograms from each of the four combinations of high or low texture score with high or low percentage dense volume is given in Fig. 1. The stronger textural properties of mammograms with high texture scores are clear in both density categories.

### Statistical analysis

Age and breast measures (mammographic density and mammographic texture scores) were determined for the first available unprocessed digital mammography examination of each woman. In addition, the number of digital screening rounds and follow-up years were determined. We described our study population by the median and interquartile range (IQR) for each of these characteristics and tested whether these characteristics were significantly different in breast cancer and non-breast cancer cases. We used the two-sample *t* test for normally distributed measures and the Mann-Whitney U test for non-normally distributed measures. Breast density measures were transformed using the natural logarithm (ln) to obtain normal distributions and Pearson correlation coefficients were determined to test correlation between breast measures and between age and breast measures.

Associations of continuous measures (per standard deviation (SD) increase, using normally distributed measures) and quartiles of density and texture scores with breast cancer risk were determined using Cox proportional hazards analyses. We calculated hazard ratios (HR) and their 95% confidence intervals (95% CI). Age was used as the underlying time scale. The entry time was defined as subject’s age at the time of the first available digital mammogram. Exit time was defined as one of the following options: (1) age at breast cancer diagnosis (event), (2) age at death (censoring), or (3) age at 2 years after the last digital mammogram performed before 1 January 2012 (censoring). The age used as the exit time was determined by the option that occurred first.

We aimed to determine whether the previously described texture score is associated with breast cancer risk and has additional value, next to volumetric mammographic density measures, in distinguishing future breast cancer cases from non-breast cancer cases. To study this, we constructed several models. First, three Cox proportional hazard models were developed with dense volume, percentage dense volume, or texture as the determinant (model 1, 2 and 3, respectively). With these models we could determine the ability of a density or texture measure alone to separate breast cancer from non-breast cancer cases. Thereafter, we constructed two additional Cox proportional hazard models. The first contained both dense volume and texture determinants (model 1a). The other model contained both percentage dense volume and texture determinants (model 2a). To determine the ability of the models to discriminate between breast cancer cases and non-cases, concordance indices (c-indices) were obtained for all models. The c-index can be seen as the fraction of “case - non-case” pairs for which the model correctly identified the breast cancer case. Across 2000 bootstrap samples, c-indices of models containing only a breast density measure (model 1 or 2) were compared to models containing both density measures and texture scores (model 1a or 2a) to test whether differences in c-indices were statistically significant.

As the density and texture scores were expected to be strongly correlated, we prevented multicollinearity from occurring in models 1a and 2a by including the residuals of the texture scores regressed on breast density instead of the texture score itself. This “residual method” is often used in the field of nutritional epidemiology [28]. Residuals were obtained by using linear regression analysis. There was no correlation between the residuals and breast density.

Additionally, two extra Cox proportional hazard models were constructed in which the residuals of breast density (dense volume for model 3a and percentage dense volume for model 3b) regressed on texture were combined with the texture score. Using these models, we could determine whether breast density measures added some distinctive power to the texture score alone.

The proportional hazards assumption was evaluated by Schoenfeld residual plots and log minus log plots, and the assumption was not violated. To examine the presence of a linear trend in HRs over the quartiles of breast measures, quartiles were added to the models as continuous variables.

Finally, in a secondary analysis we also separately determined the associations between breast measures (dense volume, percentage dense volume, and texture) and breast cancer for screen-detected and interval breast cancers. Statistical analyses were performed using SPSS version 22 and R version 3.2.0.

## Results

**Fig. 2:**
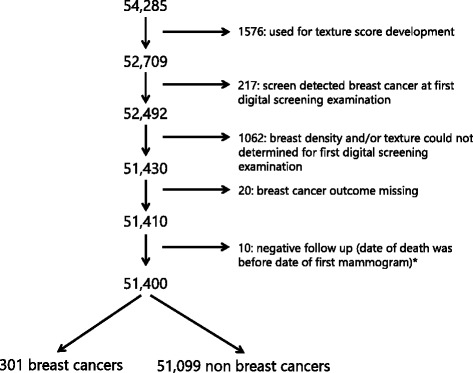
Flowchart - reasons to exclude mammograms. *We only had information on year of death and therefore we set the date of death for all women who died as 1 July in the year they died

Of the 54,285 women in our screening cohort, 898 were diagnosed with breast cancer within 2 years after their last digital screening mammogram. In the development study of the texture score used in this study, mammograms of 1576 women (both with and without breast cancer) from the aforementioned cohort were used for texture score development and therefore excluded from our analyses [24]. Next, 217 women were excluded as they were diagnosed with breast cancer as a result of their first digital screening examination and for 1062 women the breast density and/or texture scores could not be determined from the first digital screening examinations, therefore the mammograms of these women were also excluded. Finally, women were excluded for whom information on breast cancer outcome was missing (*N* = 20) and for whom the screening examination date came after the date of death (as we only had information on year of death and therefore set the date of death for all women on 1 July in the year they died) (*N* = 10). This resulted in a dataset that was used for data analysis containing 51,400 women, of whom 301 women developed breast cancer and 51,099 women did not (Fig. 2).

Characteristics of the study population are presented in Table 1. At the first available digital screening examination, the median age of women in our cohort (*N* = 51,400) was 56 years (IQR 51–63), the median dense breast volume was 57.8 cm^3^ (IQR 42.9–78.9), the percentage breast volume was 6.4% (IQR 4.8–9.8) and the texture score was 0.50 (IQR 0.48–0.53). The median total number of screening examinations (analog and digital combined) that a woman had was 5 (IQR 2–8) of which the median number of digital examinations was 2 (IQR 1–3). The median follow-up time was 4.2 years (IQ: 2.0–6.2).

**Table 1:** Characteristics of the total study population (N = 51,400) and of women with breast cancer (cases) (*N* = 301) and without breast cancer (*N* = 50,099)

Variable	Total study population Median (IQR)		Breast cancer cases Median (IQR)		Non breast cancer cases Median (IQR)		*p* value
Age (years)^a^	56	(51–63)	58	(51–63)	56	(51–63)	0.21
Digital screening rounds, number	2	(1–3)	2	(2–3)	2	(1–3)	0.05
Follow up (years)^b^	4.2	(2.0–6.2)	2.8	(1.9–4.3)	4.2	(2.0–6.2)	<0.01
Dense volume (cm^3^)^a^	57.8	(42.9–78.9)	63.9	(48.4–86.9)	57.8	(42.9–78.8)	<0.01
Percent dense volume (%)^a^	6.4	(4.8–9.8)	7.5	(5.5–7.5)	6.4	(4.8–9.8)	<0.01
Non-dense volume (cm^3^)^a^	804.9	(518.6–1183.9)	825.3	(521.8–1159.1)	804.9	(518.5–1184.0)	0.88
Total breast volume (cm^3^)^a^	866.9	(573.9–1256.7)	900.8	(592.4–1228.4)	866.8	(573.8–1256.8)	0.88
Texture score^a^	0.50	(0.48–0.53)	0.51	(0.49–0.54)	0.50	(0.48–0.53)	<0.01

^a^At first available digital screening mammogram

^b^Women were followed until breast cancer diagnosis (event), till death or till 2 years after the last available mammogram, whichever came first

Table 2 shows that age was negatively correlated with dense volume (Pearson correlation coefficient − 0.16, *p* < 0.01), percentage dense volume (− 0.29, *p* < 0.01), and texture (− 0.35, *p* < 0.01). Percentage dense volume and texture were strongly positively correlated (0.90 (*p* < 0.01)). Finally, dense volume was positively correlated with percentage dense volume (0.27, *p* < 0.01) and texture (0.20, *p* < 0.01).

**Table 2:** Pearson correlation coefficients for tests of correlation between mammographic measures and between mammographic measures and age

	Age	DV	PDV	Texture
Age	1	−0.16	−0.29	− 0.35
DV		1	0.27	0.20
PDV			1	0.90
Texture				1

Age, breast density, and texture were assessed at the first digital screening mammogram

*DV* dense volume (natural logarithm (Ln) transformed), *PDV* percent dense volume (Ln transformed)

The *p* values were statistically significant (<0.01) for all correlation coefficients

High mammographic dense volume, percentage dense breast and texture scores were all associated with a higher breast cancer risk (Table 3, model 1, 2, and 3, respectively). Women in the highest compared to the lowest quartile (Q) of dense volume had almost two times higher breast cancer risk during a median follow up of 4.2 years (Q4 vs Q1 HR 1.85, 95% CI 1.32–2.59, *p* value for trend <0.001). There were comparable results for percentage dense volume (Q4 vs Q1 HR 2.17, 95% CI 1.51–3.12, *p* value for trend <0.001). Women with a high texture pattern score had three times higher breast cancer risk than women with a low texture pattern score (Q4 vs Q1 HR 3.16, 95% CI 2.16–4.62, *p* value for trend < 0.001).

**Table 3:** The association between breast measures and breast cancer risk

Variables in the model		HR (95% CI)	HR (95% CI)	HR (95% CI)	HR (95% CI)	*p* value for trend	c-index (95% CI)
		per one SD	Q2	Q3	Q4		
Model 1	*DV*	1.32 (1.18–1.48)	1.24 (0.87–1.78)	1.53 (1.08–2016)	1.85 (1.32–2.59)	<0.001	0.56 (0.53–0.59)
Model 1a	*DV*	1.32 (1.18–1.47)	1.40 (0.97–2.01)	1.75 (1.23–2.48)	1.98 (1.41–2.79)	<0.001	0.62 (0.58–0.65)
	*Texture residuals (DV)* ^a^	1.38 (1.23–1.56)	1.68 (1.15–2.44)	2.40 (1.68–3.43)	2.69 (1.87–3.88)	<0.001	
Model 2	*PDV*	1.34 (1.20–1.50)	1.49 (1.03–2.15)	2.07 (1.46–2.96)	2.17 (1.51–3.12)	<0.001	0.58 (0.54–0.61)
Model 2a	*PDV*	1.36 (1.21–1.53)	1.50 (1.04–2.17)	2.00 (1.41–2.86)	2.15 (1.49–3.10)	<0.001	0.60 (0.57–0.63)
	*Texture residuals (PDV)* ^b^	1.27 (1.13–1.42)	1.28 (0.90–1.82)	1.69 (1.21–2.37)	1.92 (1.37–2.70)	<0.001	
Model 3	*Texture*	1.46 (1.30–1.64)	1.69 (1.15–2.50)	2.65 (1.83–3.84)	3.16 (2.16–4.62)	<0.001	0.61 (0.57–0.64)

Difference c-index model 1 and 1a, *p* < 0.001; difference c-index model 2 and 2a, *p* = 0.054

*SD* standard deviation, *Q* quartile, *DV* dense volume, *PDV* percentage dense volume

^a^Texture residuals (DV): residuals of texture pattern scores regressed on natural logarithm (Ln) transformed DV using a linear regression model

^b^Texture residuals (PDV): residuals of texture pattern scores regressed on Ln transformed PDV using a linear regression model

When the residuals of texture scores regressed on dense volume were added to the model with only dense volume (model 1a vs 1 in Table 3) the c-index increased from 0.56 (95% CI 0.53–0.59) to 0.62 (0.58–0.65). This difference was statistically significant (*p* < 0.001). When both dense volume and texture residuals were included in the model, they were both positively associated with breast cancer risk (Q4 vs Q1 HR 1.98, 95% CI 1.41–2.79, *p* value for trend <0.001 and Q4 vs Q1 HR 2.69, 95% CI1.87–3.88, *p* value for trend <0.001, respectively).

When the residuals of texture scores regressed on percentage dense volume were added to the model containing only percentage dense volume (model 2a vs 2 in Table 3) the c-index increased from 0.58 (95% CI 0.54–0.61) to 0.60 (95% CI 0.57–0.63). This difference was borderline significant (*p* = 0.054). In the model with both percentage dense volume and texture residuals (model 2a, Table 3), both breast measures showed an approximately two times higher breast cancer risk for women in the highest compared to lowest quartile (Q4 vs Q1 HR 2.15, 95% CI 1.49–3.10, *p* value for trend <0.001 and Q4 vs Q1 HR 1.92, 95% CI 1.37–2.70, *p* value for trend <0.001, respectively).

The results of the models including continuous breast density measures were in line with those including quartiles of breast measures (Table 3).

The results of model 3a (texture score in combination with the residuals of dense volume regressed on texture scores) and model 3b (texture score in combination with the residuals of *percentage* dense volume regressed on texture scores) are presented in Additional file 1: Table S1. Dense volume and percentage dense volume did not significantly improve the discriminative power in addition to the texture score (*p* = 0.076 and *p* = 0.760, respectively).

The results of analysis of the associations between breast measures and screen-detected breast cancer are presented in Additional file 2: Table S2 and Additional file 3: Table S3. The corresponding results for interval breast cancers are presented in Additional file 4: Table S4 and Additional file 5: Table S5. The associations between density or texture measures and interval breast cancer were stronger overall than the associations with screen-detected breast cancer. Both for screen-detected and for interval-detected breast cancers, the highest predictive values were for the combination of dense volume and texture models.

## Discussion

In this study, we found that the deep-learning-based texture score [24] assessed on digital mammograms was positively associated with breast cancer risk. Women in the highest quartile of texture pattern scores had approximately three times higher breast cancer risk than women in the lowest quartile. In addition, we found that the texture pattern score had additional value for the discriminatory performance next to breast density. The highest c-index was observed for the combination of dense volume with the texture score (0.62, 95% CI 0.58–0.65).

This was the first study investigating the combination of a deep-learning-based texture score method and volumetric breast density (both percent density and absolute dense volume) in relation to breast cancer risk. In a review by Gastounioti et al., studies were described in which computerized approaches with manually designed and selected texture features were used for breast cancer risk assessment on both digitized film screen and digital mammograms [19]. In some of these studies the predictive value of these texture measures was studied in combination with area-based percent density. Three of these were on digital mammograms, like our study [29–31]. The first study by Li et al. used a Bayesian artificial neural network (BANN) model and found discriminatory capacities (area under the curve (AUC)) of 0.70, 0.57, and 0.68 for texture, percent density, and the combination of both, respectively [29]. Chen et al. and Zheng et al. both used logistic regression models and found discriminatory capacities (AUC) of 0.71, 0.62, and 0.68 (Chen et al) [30] and 0.85, 0.59, and 0.86 (Zheng et al.) [31] for texture, percent density, and the combination of both, respectively. The discriminatory ability in the last study is remarkably high. As this texture score has not been externally validated, the results should be interpreted with caution. Also the studies of Li et al. and Chen et al. have not been externally validated. Additionally, in all three studies the texture score was trained and tested in the same group of cases and controls, using cross-validation techniques. The texture measure used in the current study was developed and trained in a study sample drawn from the screening cohort used in this study, also using cross-validation techniques. In this study, we, however, validated this texture score in an independent “new” dataset, as we excluded the training sample used for texture development from our cohort data.

The current texture measure was developed in a relatively large population (394 cases and 1182 controls) compared to other studies developing a texture measure [19, 24]. The use of a larger dataset reduces the chance of model overfitting. The comparable discriminative performance for texture found in the development study and the current study suggests that the degree of overfitting was only limited in the development study.

In our study, we used a cohort design, studying mammograms in a breast-cancer-free cohort and then following up for breast cancer diagnosis, for an average period of 4 years. In the previously discussed studies mammograms of the contralateral breast at the time of breast cancer diagnosis were used [29–31]. For personalized breast cancer screening, knowing which women will develop breast cancer in the future is of greater added value compared to predicting it at time of breast cancer diagnosis. In addition, by using cohort data, we were able to determine how well the texture pattern score performs in the “general screening population” instead of in a selected subset, which is the case in case-control studies.

All studies investigating the combination of texture and breast density in relation to breast cancer risk or the ability to separate breast cancer from non-breast cancer cases used area-based percent breast density measures. The most widely used quantitative are-based breast density assessment method is the semi-automatic method, Cumulus [32]. This is a very labor-intensive method to determine breast density. With the advent of digital mammography, fully automatic volumetric breast density assessment methods, like Volpara [26], have been developed. Volpara gives objective and reproducible density measurements, representing the amount of dense tissue rather than the size of the dense tissue projection as measured by area-based methods. We are the first to investigate the additional value of adding texture to both volumetric percent and absolute breast density to separate breast cancer from non-breast cancer cases.

A limitation of our study is that as far as potential confounders are concerned, we only had information about age. We made use of anonymized routinely collected screening data and the Dutch screening program, like many other screening programs, does not collect any information on risk factors. In studies where adjustment for breast cancer risk factors, in particular body mass index, was possible, this usually led to slightly higher risk estimates for percent density [14, 22]. The association between absolute dense volume and breast cancer risk is hardly influenced by adjustment for body mass index (BMI) [33, 34]. Despite the absence of information on BMI or other breast cancer risk factors, we think that our study provides useful information as to the additional value of adding texture characteristics to breast density estimates. In many screening programs there is either no information or no extensive information available on risk factors other than age, and breast tissue characteristics can be relatively easily obtained from the mammograms. Another limitation is the fact that the texture pattern score in this study was trained on mammograms of women from the same screening population. Despite the fact that mammograms that were used for texture training were excluded from our study population, one might expect that the mammograms that were used for texture training were more similar to the mammograms in our study population as compared to other breast cancer screening populations in the world. Therefore, the performance of this texture pattern score should also be externally validated in other screening populations.

Strengths of this study are the automatically measured density and texture scores, as they give objective and reproducible results. In addition, these are high-throughput methods which make them suitable for screening practice. Finally, the ability of breast density and texture to discriminate non-breast cancer from breast cancer cases in a population-based breast cancer screening cohort resembles the performance of these measures in real screening practice, probably better than when using a case-control study.

## Conclusions

Deep-learning-based texture pattern scores measured automatically on digital mammograms were shown to be related to breast cancer risk. Additionally, texture pattern scores statistically significantly improved the discriminatory performance in addition to absolute dense volume. Therefore, texture measures in addition to density may be taken into account in the development of more personalized breast cancer screening.

## Additional files

Additional file 1: **Table S1.** Texture measures in combination with breast density and breast cancer risk. (DOCX 16 kb)

Additional file 2: **Table S2.** The association between breast measures and screen-detected breast cancer risk. (DOCX 17 kb)

Additional file 3: **Table S3.** Texture measures in combination with breast density and screen-detected breast cancer risk. (DOCX 16 kb)

Additional file 4: **Table S4.** The association between breast measures and interval breast cancer risk. (DOCX 17 kb)

Additional file 5: **Table S5.** Texture measures in combination with breast density and interval breast cancer risk. (DOCX 15 kb)

## Abbreviations

AUC: Area under the curve
BANN: Bayesian artificial neural network
CC: Craniocaudal
CI: Confidence interval
DM: Digital mammography
DV: Dense volume
HR: Hazard ratio
IQR: Interquartile range
Ln: Natural logarithm
MLO: Mediolateral oblique
MRI: Magnetic resonance imaging
PDV: Percentage dense volume
Q: Quartile
SD: Standard deviation
US: Ultrasound
