# Supplementary material for: The combined effect of mammographic texture and density on breast cancer risk: a cohort study
Source: Breast Cancer Res. 2018 May 2;20:36. doi: 10.1186/s13058-018-0961-7 (PMC5932877; doi:10.1186/s13058-018-0961-7)
Supplement: Supplementary file 1 — Table S1. Texture measures in combination with breast density and breast cancer risk. (DOCX 16 kb) [file 13058_2018_961_MOESM1_ESM.docx]

**Additional file 1: Table S1. Texture measures in combination with breast density and breast cancer risk**

| **Variables in the model** | | **HR (95% CI)** |  | **HR (95% CI)** | **HR (95% CI)** | **HR (95% CI)** | **p-value for trend** | **C-index (95% CI)** |
| --- | --- | --- | --- | --- | --- | --- | --- | --- |
|  |  | **per one SD^*^** |  | **Q2** | **Q3** | **Q4** |  |  |
| **Model 3** | ***Texture*** | 1.46 (1.30-1.64) |  | 1.69 (1.15-2.50) | 2.65 (1.83-3.84) | 3.16 (2.16-4.62) | <0.001 | 0.61 (0.57-0.64) |
| **Model 3a** | ***Texture*** | 1.45 (1.29-1.63) |  | 1.82 (1.23-2.69) | 2.85 (1.97-4.14) | 3.25 (2.22-4.76) | <0.001 | 0.62 (0.59-0.66) |
|  | ***DV residuals (Texture)^1^*** | 1.23 (1.10-1.37) |  | 1.33 (0.93-1.89) | 1.59 (1.13-2.23) | 1.69 (1.20-2.36) | 0.003 |  |
| **Model 3b** | ***Texture*** | 1.46 (1.30-1.65) |  | 1.64 (1.11-2.43) | 2.56 (1.77-3.72) | 3.11 (2.13-4.55) | <0.001 | 0.61 (0.58-0.65) |
|  | ***PDV residuals (Texture)^2^*** | 0.93 (0.83-1.04) |  | 1.00 (0.73-1.36) | 0.78 (0.56-1.09) | 0.85 (0.62-1.18) | 0.111 |  |

*SD: standard deviation; Difference C-index model 3 & 3a: p=0.076; Difference C-index model 3 & 3b: p=0.760.
1. DV residuals (Texture): Residuals of ln transformed dense volume regressed on texture pattern scores using a linear regression model.
2. PDV residuals (Texture): Residuals of ln transformed percentage dense volume regressed on texture pattern scores using a linear regression model.
