# Supplementary material for: The combined effect of mammographic texture and density on breast cancer risk: a cohort study
Source: Breast Cancer Res. 2018 May 2;20:36. doi: 10.1186/s13058-018-0961-7 (PMC5932877; doi:10.1186/s13058-018-0961-7)
Supplement: Supplementary file 2 — Table S2. The association between breast measures and screen-detected breast cancer risk. (DOCX 17 kb) [file 13058_2018_961_MOESM2_ESM.docx]

**Additional file 2: Table S2. The association between breast measures and screen detected breast cancer risk**

| **Variables in the model** | | **HR (95% CI)** |  | **HR (95% CI)** | **HR (95% CI)** | **HR (95% CI)** | **p-value for trend** | **C-index** |
| --- | --- | --- | --- | --- | --- | --- | --- | --- |
|  |  | **per one SD^*^** |  | **Q2** | **Q3** | **Q4** |  |  |
| **Model 1** | ***DV*** | 1.21 (1.03-1.41) |  | 1.38 (0.87-2.20) | 1.64 (1.05-2.57) | 1.57 (0.99-2.50) | 0.041 | 0.537 |
| **Model 1a** | ***DV*** | 1.22 (1.04-1.41) |  | 1.50 (0.94-2.39) | 1.79 (1.11-2.81) | 1.64 (1.03-2.63) | 0.029 | 0.583 |
|  | ***Texture residuals (DV)^1^*** | 1.23 (1.05-1.44) |  | 1.17 (0.74-1.84) | 1.85 (1.21-2.84) | 1.77 (1.11-2.83) | 0.005 |  |
| **Model 2** | ***PDV*** | 1.13 (0.96-1.33) |  | 1.24 (0.80-1.92) | 1.60 (1.04-2.46) | 1.41 (0.88-2.26) | 0.071 | 0.544 |
| **Model 2a** | ***PDV*** | 1.14 (0.96-1.35) |  | 1.25 (0.81-1.94) | 1.55 (1.01-2.38) | 1.40 (0.87-2.26) | 0.091 | 0.583 |
|  | ***Texture residuals (PDV)^2^*** | 1.31 (1.11-1.54) |  | 1.49 (0.94-2.37) | 1.82 (1.15-2.86) | 2.20 (1.39-3.48) | <0.001 |  |
| **Model 3** | ***Texture*** | 1.27 (1.08-1.50) |  | 1.41 (0.90-2.23) | 1.97 (1.27-3.07) | 1.96 (1.21-3.18) | 0.001 | 0.574 |

*SD: standard deviation, Q: quartile;
1. Texture residuals (DV): Residuals of texture pattern scores regressed on ln transformed dense volume using a linear regression model.
2. Texture residuals (PDV): Residuals of texture pattern scores regressed on ln transformed percentage dense volume using a linear regression model.
