# Supplementary material for: The combined effect of mammographic texture and density on breast cancer risk: a cohort study
Source: Breast Cancer Res. 2018 May 2;20:36. doi: 10.1186/s13058-018-0961-7 (PMC5932877; doi:10.1186/s13058-018-0961-7)
Supplement: Supplementary file 3 — Table S3. Texture measures in combination with breast density and screen-detected breast cancer risk. (DOCX 16 kb) [file 13058_2018_961_MOESM3_ESM.docx]

**Additional file 3: Table S3. Texture measures in combination with breast density and screen detected breast cancer risk**

| **Variables in the model** | | **HR (95% CI)** |  | **HR (95% CI)** | **HR (95% CI)** | **HR (95% CI)** | **p-value for trend** | **C-index** |
| --- | --- | --- | --- | --- | --- | --- | --- | --- |
|  |  | **per one SD^*^** |  | **Q2** | **Q3** | **Q4** |  |  |
| **Model 3** | ***Texture*** | 1.27 (1.08-1.50) |  | 1.41 (0.90- 2.23) | 1.97 (1.27-3.07) | 1.96 (1.21-3.18) | 0.001 | 0.574 |
| **Model 3a** | ***Texture*** | 1.27 (1.08-1.50) |  | 1.50 (0.95-2.36) | 2.10 (1.35-3.27) | 2.06 (1.27-3.34) | 0.001 | 0.583 |
|  | ***DV residuals (Texture)^1^*** | 1.16 (1.00-1.35) |  | 1.52 (0.96-2.40) | 1.71 (1.09-2.68) | 1.47 (0.92-2.36) | 0.140 |  |
| **Model 3b** | ***Texture*** | 1.27 (1.07-1.50) |  | 1.33 (0.84-2.10) | 1.85 (1.18-2.88) | 1.91 (1.18-3.10) | 0.002 | 0.583 |
|  | ***PDV residuals (Texture)^2^*** | 0.83 (0.71-0.98) |  | 0.82 (0.55-1.23) | 0.69 (0.45-1.05) | 0.66 (0.43-1.02) | 0.027 |  |

*SD: standard deviation; Q: Quartile
1. DV residuals (Texture): Residuals of ln transformed dense volume regressed on texture pattern scores using a linear regression model.
2. PDV residuals (Texture): Residuals of ln transformed percentage dense volume regressed on texture pattern scores using a linear regression model.
