# Supplementary material for: The combined effect of mammographic texture and density on breast cancer risk: a cohort study
Source: Breast Cancer Res. 2018 May 2;20:36. doi: 10.1186/s13058-018-0961-7 (PMC5932877; doi:10.1186/s13058-018-0961-7)
Supplement: Supplementary file 4 — Table S4. The association between breast measures and interval breast cancer risk. (DOCX 17 kb) [file 13058_2018_961_MOESM4_ESM.docx]

**Additional file 4: Table S4. The association between breast measures and interval cancer breast cancer risk**

| **Variables in the model** | | **HR (95% CI)** |  | **HR (95% CI)** | **HR (95% CI)** | **HR (95% CI)** | **p-value for trend** | **C-index** |
| --- | --- | --- | --- | --- | --- | --- | --- | --- |
|  |  | **per one SD^*^** |  | **Q2** | **Q3** | **Q4** |  |  |
| **Model 1** | ***DV*** | 1.47 (1.24-1.74) |  | 1.05 (0.59-1.88) | 1.39 (0.81-2.38) | 2.16 (1.31-3.55) | <0.001 | 0.61 |
| **Model 1a** | ***DV*** | 1.45 (1.23-1.70) |  | 1.23 (0.69-2.19) | 1.68 (0.98-2.90) | 2.39 (1.44-3.95) | <0.001 | 0.67 |
|  | ***Texture residuals (DV)^1^*** | 1.58 (1.33-1.89) |  | 3.79 (1.79-8.02) | 4.73 (2.28-9.81) | 6.08 (2.95-12.52) | <0.001 |  |
| **Model 2** | ***PDV*** | 1.60 (1.36-1.88) |  | 2.32 (1.15-4.68) | 3.62 (1.86-7.04) | 4.30 (2.22-8.34) | <0.001 | 0.64 |
| **Model 2a** | ***PDV*** | 1.64 (1.39-1.94) |  | 2.32 (1.15-4.68) | 3.49 (1.79-6.80) | 4.26 (2.19-8.26) | <0.001 | 0.65 |
|  | ***Texture residuals (PDV)^2^*** | 1.23 (1.04-1.46) |  | 1.03 (0.60-1.78) | 1.53 (0.92-2.52) | 1.61 (0.98-2.65) | 0.019 |  |
| **Model 3** | ***Texture*** | 1.71 (1.44-2.04) |  | 2.89 ( 1.29-6.46) | 5.40 (2.53-11.53) | 7.38 (3.47-15.74) | <0.001 | 0.65 |

*SD: standard deviation, Q: quartile;
1. Texture residuals (DV): Residuals of texture pattern scores regressed on ln transformed dense volume using a linear regression model.
2. Texture residuals (PDV): Residuals of texture pattern scores regressed on ln transformed percentage dense volume using a linear regression model.
