# Supplementary material for: The combined effect of mammographic texture and density on breast cancer risk: a cohort study
Source: Breast Cancer Res. 2018 May 2;20:36. doi: 10.1186/s13058-018-0961-7 (PMC5932877; doi:10.1186/s13058-018-0961-7)
Supplement: Supplementary file 5 — Table S5. Texture measures in combination with breast density and interval breast cancer risk. (DOCX 15 kb) [file 13058_2018_961_MOESM5_ESM.docx]

**Additional file 5: Table S5. Texture measures in combination with breast density and interval cancer breast cancer risk**

| **Variables in the model** | | **HR (95% CI)** |  | **HR (95% CI)** | **HR (95% CI)** | **HR (95% CI)** | **p-value for trend** | **C-index** |
| --- | --- | --- | --- | --- | --- | --- | --- | --- |
|  |  | **per one SD^*^** |  | **Q2** | **Q3** | **Q4** |  |  |
| **Model 3** | ***Texture*** | 1.71 (1.44-2.04) |  | 2.89 ( 1.29-6.46) | 5.40 (2.53-11.53) | 7.38 (3.47-15.74) | <0.001 | 0.65 |
| **Model 3a** | ***Texture*** | 1.69 (1.42-2.01) |  | 3.15 (1.41-7.07) | 5.89 (2.75-12.62) | 7.46 (3.50-15.91) | <0.001 | 0.67 |
|  | ***DV residuals (Texture)^1^*** | 1.31 (1.11-1.54) |  | 1.08 (0.62-1.88) | 1.43 (0.85-2.41) | 1.91 (1.78-3.11) | 0.008 |  |
| **Model 3b** | ***Texture*** | 1.71 (1.44-2.04) |  | 2.89 (1.29-6.49) | 5.42 (2.53-11.61) | 7.35 (3.44-15.68) | <0.001 | 0.65 |
|  | ***PDV residuals (Texture)^2^*** | 1.03 (0.88-1.22) |  | 1.29 (0.80-2.09) | 0.93 (0.55-1.56) | 1.18 (0.72-1.91) | 0.962 |  |

*SD: standard deviation;
1. DV residuals (Texture): Residuals of ln transformed dense volume regressed on texture pattern scores using a linear regression model.
2. PDV residuals (Texture): Residuals of ln transformed percentage dense volume regressed on texture pattern scores using a linear regression model.
